# Is Searching for Martian Life a Priority for the Mars Community?

**DOI:** 10.1089/ast.2017.1772

**Published:** 2018-02-01

**Authors:** Alberto G. Fairén, Victor Parro, Dirk Schulze-Makuch, Lyle Whyte

**Affiliations:** ^1^ Centro de Astrobiología (CSIC-INTA), Madrid, Spain.; ^2^ Department of Astronomy, Cornell University, Ithaca, New York, USA.; ^3^ Center of Astronomy and Astrophysics, Technical University Berlin, Berlin, Germany.; ^4^ School of the Environment, Washington State University, Pullman, Washington, USA.; ^5^ Department of Natural Resource Sciences, McGill University, Ste-Anne-de-Bellevue, Canada.

We understand and respect the points raised by Rummel and Conley (2017) in response to our initial Forum Article (Fairén *et al.,* 2017), which we acknowledge are informed and literate. Unfortunately, they are also unconvincing. Their comments clearly illustrate why we are not searching for life on Mars today and why we haven't done so during the last decades. Hereafter, we respond point by point to their remarks. We examine with special attention Rummel and Conley's (2017) statement that “the Mars community is not convinced that a mission to attempt detection of extant martian life is a high priority,” because in our view this sentence reflects an alarming disconnection between the opinions of the NASA Planetary Protection Officers and the actual priorities, goals, and desires of the Mars community. Note that, to address their comments specifically, we cite them as they were presented by Rummel and Conley (2017). We conclude that searching for life on Mars before it is too late requires a reassessment of current policies.

## 1. Planetary Protection Policies and the Ultimate Goal of Mars Exploration

In their introduction, Rummel and Conley (2017) stated that “COSPAR's planetary protection policy is maintained as an international consensus standard for spacecraft cleanliness” and “COSPAR Planetary Protection Policy has included explicit principles and guidelines relating to human Mars missions since 2008.” We agree. One of the points of our paper was precisely that these guidelines need to be reconsidered and updated, because some accepted popular concepts are either outdated or simply wrong (Box 1). Our initial Forum Article (Fairén *et al.,* 2017), while based on robust scientific rationale, was also meant to be provocative and push the scientific community to address the most important scientific question driving Mars exploration; that is, has life occurred on Mars, in the past or at present, and how can we best answer this question with future Mars missions?

Box 1: Careful with Widespread Fallacious Analogies **1. We brought smallpox to the New World when the Europeans came over, and they took home syphilis.**

Of course smallpox and syphilis travelled between human populations living in temperate latitudes, but that situation is irrelevant to Mars exploration. In any analogy addressing possible biological exchange between Earth and Mars, it is unavoidable considering the absolutely contrasting environments of both planets. A more accurate analogy could be the following: if we bring 12 Asian tropical parrots to the rainforest in Venezuela, in 10 years we'll very likely have an invasion of Asian parrots in South America; but if we bring the same 12 Asian parrots to Antarctica, in 10 hours we'll have 12 dead parrots.

**2. Would you let a doctor do surgery on you who didn't sterilize the instruments beforehand?**

The level of cleanliness we achieve in our spacecraft assembly rooms will happen naturally anyway (and even more) in a mission to Mars, because all the microorganisms that we kill in spacecraft assembly rooms will be killed anyway on Mars (and then some), and especially during the trip to Mars, with the result that they won't be active even 1 hour on the surface of Mars. On the contrary, if a doctor doesn't clean the instruments before surgery, microorganisms will be there during the operation, and they may infect us, because the environment in the OR won't kill them.

### 2. Human versus Robotic Exploration Challenges

Rummel and Conley (2017) argued that “contamination from poorly prepared robotic missions could spread as easily as contamination associated with human missions.” This is incorrect. Robotic missions, even if they are not cleaned at all, will carry on them only the bioburden that they have when they leave Earth—that's it. Once the spacecraft is outside Earth and the microorganisms on board start dying, there will be no replacement for the dead microbes. And for those microorganisms still surviving the trip, there will be no protection or food, so the harsh environment of interplanetary travel and on the surface of Mars will kill them all eventually. On the other hand, human missions will keep microbes alive as long as humans are alive, because the mission will be designed to keep the astronauts alive, which will keep the microbes alive, too, perhaps even allowing them to reproduce.

In the end, a spacesuit or a human habitat on Mars will be engineered to preserve life inside and over a long period of time, while a rover or lander is intended to be lifeless. In addition, the microbial load on a human mission will be inherently orders of magnitude higher in number and diversity than on a robotic mission, as Rummel and Conley (2017) recognized in their piece: “it is anticipated that future human missions to Mars will increase the amount of biological and organic contamination that might be distributed on that planet,” and “human missions will inevitably bring a large population of Earth microbes along with them.” This was also pointed out by Conley and Rummel (2010) in that “some degree of forward contamination associated with human astronaut explorers is inevitable.” The contamination risks between robotic and manned missions are simply not comparable, because “the initial population size and composition (*i.e.,* the bioload)” of a mission will determine “the probability of microbes surviving either natural or human-mediated transfer” (Fajardo-Cavazos *et al.,* 2007).

## 3. Special Regions

The argument of Rummel and Conley (2017) regarding Special Regions is summarized in the following passages from their article:

“Are Special Regions the best places to search for martian life?”

“… there are currently no parameters explicitly defining [a place known to host martian life].”

“… no confirmed Special Regions have yet been shown to exist on Mars.”

But if Special Regions are not *the best places* for martian life to exist, what exactly are we protecting on Mars with the planetary protection policies? In fact, why are we protecting any place on Mars if there are *no confirmed Special Regions?*

These authors' contradictory arguments become even more evident given that they previously stated that “the concept of Mars Special Regions plays a key role in protecting astrobiology on the planet.” How is this possible if “no confirmed Special Regions have yet been shown to exist on Mars”? That is, how can something nonconfirmed to exist play a “key role” in anything? In addition, how can Special Regions protect astrobiology on Mars if they are not “the best places” to host martian life?

The authors continue their line of arguments, stating that “we keep discovering life growing in extreme conditions on Earth that resemble conditions on Mars.” This comment raises two immediate concerns. Firstly, where exactly on Mars are those places that resemble Earth extreme localities? Could they be considered Special Regions? Note that Rummel and Conley (2017) also stated that “we keep discovering conditions on Mars that are more similar to inhabited environments on Earth, which is where the concept of Special Regions initially came from.” This would be in direct contradiction to their previous argument that it is not clear whether there exist confirmed Special Regions on Mars. Secondly and more importantly, we addressed this point carefully in our initial article: there is no study to date demonstrating that an Earth microbe could produce a viable microbial ecosystem under the complete range of conditions on the surface of Mars. We have numerous studies pointing to resistance to some of those conditions, separately, but none considering the full range of martian surface conditions, including temperature, pressure, atmospheric composition, radiation, humidity, oxidizing regolith, and others, all at the same time and in combination. In fact, “in the majority of Mars simulation studies, parameters (pressure, temperature, UV, etc.) have been applied either singly or in at most a combination of two or three. Thus, at present it is unknown how microorganisms respond to the complete suite of martian environmental conditions applied simultaneously” (Rummel *et al.,* 2014).

Rummel and Conley (2017) subsequently elaborated on their advice to prevent the possibility that the Curiosity rover could explore recurrent slope lineae (RSL): “It is probable that a mission-ending accident would be of concern to rover operators for other reasons.” If Curiosity approaches RSL, this, as a first step, could provide sufficient resolution to return invaluable information about the nature of the RSL and possibly bring the debate about whether the RSL are briny or not to a conclusion (see Ojha *et al.,* 2015, and Bhardwaj *et al.,* 2017; and the contrasting view in Edwards and Piqueux, 2016, and Dundas *et al.,* 2017), with no substantially different problems in trafficability than those occurring when crossing a sand dune, for example. And that's assuming RSL are formed by briny outflows that could create a crust that might only appear to be solid and therefore are potentially dangerous for traversing; if RSL are not briny outflows, then there are no potential salt crusts that the rover could hypothetically break through. Indeed, please note that our arguments regarding RSL exploration by Curiosity are equally applicable to any other rover if it turns out that the indications of dark slope streaks with the temporal behavior interpreted as evidence for water in Gale Crater are not conclusive.

Also, we note that Rummel *et al.* (2014) stated that RSL “meet the criteria to be treated as Special Regions.” Therefore, on one hand they state that “no confirmed Special Regions have yet been shown to exist on Mars,” but on the other hand they require that RSL should be “treated” as if they were Special Regions. This distinction is blurry to say the least.

In the end, Rummel and Conley (2017) acknowledged that “the best of the candidates” to be Special Regions “currently under consideration would be unreachable with current mission capabilities (*e.g.,* deep subsurface aquifers).” This means that the best candidates to be Special Regions on Mars are hypothetical, nonconfirmed, and unreachable, bringing our initial question to the table again: What exactly are we protecting with planetary protection policies on Mars?

## 4. Astrobiological Exploration

Rummel and Conley (2017) responded to our assessment (Fairén *et al.,* 2017) that to clean spacecraft to the levels required for Special Regions access is very expensive by briefly concluding that it is not. This is in contradiction to previous and current analyses by several groups, suggesting that (i) applying a microbial bioload reduction at least to the surface of Mars landed missions “could be unnecessarily expensive, depending on the scientific objectives of the mission” (Rummel, 2009); (ii) the exploration of Special Regions will entail that “the cost of future Mars missions may increase significantly” because “missions that target these areas are required to comply with very expensive sterilization procedures” (Martín-Torres and Zorzano, 2017); or (iii), for sample return, “most spacecraft designers believe it would be extraordinarily expensive to build a spacecraft with modern avionics that could be heat sterilized the way Viking was” (Mattingly *et al.,* 2002).

In any case, if we assume the premise that it is not so costly or difficult, then the question immediately arises as to why we are not investigating Special Regions today. This is not a rhetorical question; if it is not budget constraints, what is the reason that we have not conducted true life-seeking missions on Mars since Viking? Rummel and Conley (2017) claimed that the Mars community has no interest in such missions. We doubt that very much, and we will return to that point in detail at the end of this article, because we feel that this is at the very core of this discussion.

Rummel and Conley (2017) added that we should ensure that “we don't instead find the Earth life we brought with us.” This is an outdated argument, largely addressed in our previous articles (Fairén and Schulze-Makuch, 2013; Fairén *et al.,* 2017) and by many other researchers before (see references in Fairén *et al.,* 2017, and also, *i.e.,* La Duc *et al.,* 2017). Half of our 2017 article was about this question, in which we argued that this is not such an overwhelming problem as it was in the past; in fact, it is solvable. We are clever enough to recognize martians, if they exist.

## 5. Recognizing the Martians

Rummel and Conley (2017) questioned whether “nucleic acid sequence comparison can distinguish definitively between martian and Earth life.” Note that when we mentioned nucleic acids in our original article (Fairén *et al.,* 2017), we did so after carefully disclaiming that our arguments thereafter would only apply *if* (a big if) martian life is genetically similar to Earth's. Rummel and Conley ignored our disclaimer and then elaborated on the possibility that martian and Earth life would be genetically different; we do not object to those comments.

But later in their response, Rummel and Conley alerted about the possibility of lateral gene transfer between terrestrial and martian biospheres “if Earth life and martian life are related, or if martian life is unrelated but still uses DNA/RNA.” This is in blatant contradiction to their previous argument that “there is a very real possibility that extraterrestrial life does not possess either RNA or DNA, which would make any question of sequence comparison irrelevant.” Given that these authors used this argument before to discredit our points about identifying life on Mars, Rummel and Conley (2017) should not have used the reverse argument. Either there could be genetic relation or similarity, and then our biochemical techniques are valid to distinguish between martian and Earth life, and there is a danger of lateral gene transfer; or there is no genetic relation. Using one argument on one side of the issue and then the opposite argument on other side of the same issue, however it fits, is not acceptable in a scientific debate.

To address the core of this issue, it should be said that there are indeed plenty of other strategies proposed with which to search for extant life on Mars, and most of them do not involve DNA/RNA examination (*i.e.,* Parro *et al.,* 2011; Azua-Bustos and Vega-Martínez, 2013; McKay *et al.,* 2013; Schulze-Makuch *et al.,* 2013; Court *et al.,* 2014; Woolman *et al.,* 2015; Benner, 2017; Carr *et al.,* 2017; Mojarro *et al.,* 2017). Incidentally, these works speak volumes about the interest of the Mars community in searching for present life on Mars. And we also mentioned in our article that we “need parallel analyses for complex and polymeric sugars, lipids, and peptides” as examples of different strategies.

Finally, in our initial Forum Article (Fairén *et al.,* 2017), we explained that we can certainly distinguish between martian and Earth life because we can identify and control the diversity and quantity of microbial populations in our clean rooms, and therefore the microorganisms potentially travelling in our spacecraft can be recognized. Conversely, the landing of manned missions will alter the martian surface forever, because “human missions will carry microbial populations that will vary in both kind and quantity, and it will not be practicable to specify all aspects of an allowable microbial population or potential contaminants at launch” (Conley and Rummel, 2010).

## 6. Human Exploration

Rummel and Conley (2017) asked “why should robots be allowed to contaminate Mars, before humans get there” and further indicated that we did not justify our alleged contention that exploration with dirty robots, now, is preferable to the possibility that “later contamination will be spread by human exploration.” These authors also asserted that we argued “it would be advantageous to contaminate Special Regions with robots, now, so that humans won't get to be the first to do it later.” Here, Rummel and Conley (2017) seem to have fallen onto straw man arguments, because we never purported a *contaminate now better than later* strategy, nor did we indicate that contamination could be “advantageous” in any scenario or in any way. What we asserted basically was that we are not really contaminating now, but we will later, so please be proactive and act now. And we uphold our claim here, noting that there is growing evidence for a bactericidal martian regolith (*i.e.,* Cockell *et al.,* 2000; Schuerger and Nicholson, 2006; Fox-Powell *et al.,* 2016; Wadsworth and Cockell, 2017, and references therein).

Our point, overall, was that it would be very sad to see in the nightly news, 20 or 30 years from now, how a waste disposal system broke in a private- or state-managed martian human base, while space agencies in the West are still busy discussing the level of cleanliness required to allow our rovers to investigate a Special Region in search of extant life. As indicated by Rummel *et al.* (2014), “it will be impossible for all human-associated processes and operations to be conducted within entirely closed systems.” The point of our Forum Article was precisely to highlight the need to take Mars exploration in its entirety into account and take action now to avoid possible future regrets.

## 7. Harmful Terrestrial Organisms Overlooked?

Rummel and Conley (2017) claimed that we did not address the possibility that Earth organisms can be harmful for future astronauts. First, this point, which they put forth as a “fact,” is not currently supported by any robust science. They argued that non-harmful Earth microbes carried today could “mutate in the space environment” in just a few decades and turn out to be harmful for future astronauts (indeed, carrying already harmful microbes such as the flu or Ebola is obviously dangerous in the first place and therefore is not the question here). The authors seem to ignore the basic fact of pathogenicity that, for a non-harmful microbe to become a pathogen to a host, molecular interactions and coevolution with that host are a prerequisite (*e.g.,* May and Anderson, 1990; Woolhouse *et al.,* 2002). Population genetics models inform that a microbe cannot mutate into a pathogen for humans while isolated on Mars (Dybdahl *et al.,* 2014). The authors also ignore that such a process would take time, because evolution is not apparent in just a few decades, and the hypothetical mutating microbes from Earth would need to maintain themselves and spread on Mars. And second, the authors are very specific about potentially mutating microbes traveling on future manned missions and not on our unmanned robots today, so their claim seems to reinforce the arguments in our initial article (Fairén *et al.,* 2017), which are that future contamination carried along by astronauts will be far more dangerous than current risks associated with robots.

Their discussion then shifts to “self-calcifying crack-closing microbes used as a concrete additive” on Earth. But could these or any other of the “certain organisms” mentioned by Rummel and Conley (2017), citing Jonkers *et al.* (2010), which are aerobic heterotrophs, thrive (*i.e.,* reproduce and evolve) under today's martian conditions? The answer is no. If such Earth organisms were introduced into an underground martian environment, they would be rendered lifeless in a time measured in minutes or less.

## 8. Backward Contamination and Sample Return

Rummel and Conley (2017) stated that “the current COSPAR Planetary Protection Policy has been central in efforts to protect against the backward contamination of Earth.” Indeed, we did not address backward contamination in our initial paper (Fairén *et al.,* 2017), but we are happy to state our position on this different debate here.

If martian microbes exist, they have been arriving at Earth for billions of years via meteorites, given that the trip from Mars to Earth is easier than from Earth to Mars, and it has been estimated that this happens 10 times more frequently (Rummel and Conley 2017, citing Mileikowsky *et al.,* 2000). Therefore, it is possible that the putative martians have already arrived on Earth.

In any case, we agree that the rules for backward contamination should remain in place. After all, we still don't know whether returning samples could endanger humanity and the terrestrial biosphere if there is life on Mars. Relaxing the forward contamination rules and allowing a dedicated search for life on Mars now would actually assist in understanding the actual risks of returning samples from Mars in the future, as we will have a better idea whether there is life on Mars or not, and what robots or astronauts might bring back to Earth, consequently increasing the safety of Earth's biosphere. Rummel and Conley (2017) have a very valid point when they state that “if an astronaut exploring Mars is likely to run into martian organisms, that fact should be well understood before landing there.” We heartily agree, and the same applies for sample return. The only way to know whether there are martians or not would be to send robots to Mars now and explore the planet astrobiologically, before bringing back samples to Earth or sending astronauts to Mars.

## 9. Rummel and Conley's (2017) Conclusions

Rummel and Conley (2017) concluded that “there is still time to explore Mars properly.” This was exactly the point of our initial article (Fairén *et al.,* 2017): let's explore Mars properly now, while there is still time. The question, though, is what “properly” means. Does “properly” include Special Regions? If it does, this contradicts their argument about the unclear nature of the Mars Special Regions; and if it does not, it would not be done “properly.”

Rummel and Conley also argued that “it is important that missions looking for indigenous life on Mars do so with the most effective contamination control measures available.” It is not clear what the purpose behind this sentence was, because there currently are no “missions looking for indigenous life on Mars”: that's precisely the problem we are highlighting, and it was the main point of our initial article (Fairén *et al.,* 2017).

Finally, they claim that our approach is “self-limiting and does nothing for future human exploration.” The problem is that there are important shortcomings in the current approach, the one defended by Rummel and Conley, as the existing policies inhibit the search for life on Mars. If policies don't change, we will not have a true life-seeking mission before astronauts get there.

## 10. Our Conclusion

As we have shown in this reply, the arguments in Rummel and Conley (2017) are based on previous perceptions that are generally outdated. No new ideas are offered in their reply to our original Forum Article (Fairén *et al.,* 2017), and as we have highlighted here, their reply contains a significant number of internal contradictions. Therefore, we maintain our well-supported claim that the current robotic exploration of Mars will have little (if any) impact on potential martian biospheres or on our efforts for searching for active life on Mars, because (i) the microbial burden carried by unmanned robots is minimal and not renewable and, most importantly, known and identifiable; (ii) the martian surface is bactericidal in nature; and (iii) we know how to distinguish an Earth microorganism from potential martians.

The inevitable conclusion is that we need to resume a dedicated robotic search for life on Mars as soon as possible, before manned missions reach the planet and it becomes too late. This requires a reassessment of the current planetary protection policies in Mars exploration. MER- and MSL-like cleanliness levels should be enough to allow a robot to search for life on Mars, especially given that, after the interplanetary trip and just one single sol on the surface of Mars receiving direct sunlight, 99.999% of the most radiation-resistant bacterial strains known to exist in clean rooms would be inactivated or killed, including spores (Khodadad *et al.,* 2017). Therefore, any possible spacecraft touching down on the surface of Mars with the mission of searching for life will be as biologically clean (and maybe even more) as the Viking probes were when they left Earth.

## 11. A Proposal to Move This Discussion Forward: Let's Check the Priorities of the Mars Community

For us, the most perplexing contention of Rummel and Conley's response to our Forum Article was their comment purporting an alleged lack of interest in an astrobiological mission to Mars. Rummel and Conley (2017) stated that “there is no consensus within the Mars community for conducting missions that might detect extant Mars life” and “the Mars community is not convinced that a mission to attempt detection of extant Mars life is a high priority.” Here, the authors do not justify their contention.

This is certainly not how the Mars community feels. We uphold that there is nothing more appealing to the Mars community (and exciting for planetary research enthusiasts worldwide) than the prospect of finding life on Mars. The search for life on Mars is one of the most fascinating scientific endeavors of our time. And interest in the search for life goes beyond the Mars science community, which is clear by the amount of press and media coverage missions to Mars receive.

The Mars community, including NASA and ESA, always points to life detection as a number-one priority in Mars exploration (*i.e.,* Grossman, 2013; McKay *et al.,* 2013; Heldmann *et al.,* 2014; King, 2015; Vago *et al.,* 2015; Gordon and Sephton, 2016; Levin and Straat, 2016; Ehlmann *et al.,* 2017; Hubbard, 2017; Niles *et al.,* 2017; Smith *et al.,* 2017; Xie *et al.,* 2017; Hand, 2018; Michalski *et al.*, 2018). This position is also well summarized in (i) the motivating goal #1.B of the Mars Exploration Program: “Determine if environments with high potential for *current* habitability and expression of biosignatures contain evidence of *extant* life” (MEPAG, 2015; italics added); (ii) the fact that the NASA Astrobiology Institute was established shortly after the possible detection of fossilized life on Mars (McKay *et al.,* 1996); (iii) the initiative that the Chairs of the Mars Habitability Session at the 2010 Astrobiology Science Conference took to ask for adding a life-detection mission into the NASA Decadal Survey, which included a petition signed by more than 130 scientists, most of them still active in the Mars science community (Schulze-Makuch and Davila, 2010); and (iv) the debate held recently in this journal (the leading journal in the field) regarding the search for life on Mars (Schulze-Makuch *et al.,* 2015).

At the same time, all Mars missions are put forward as relevant for life detection. For example, NASA's Science Goal 1 for Mars exploration states: “On Mars, we will therefore search for evidence of life in areas where liquid water was once stable, and below the surface where it still might exist *today*” (https://mars.nasa.gov/programmissions/science/goal1; italics added). ESA argues in the same way that a “basic question” to answer in Mars exploration is whether “was there ever, or *is there still*, life on Mars” and that spacecraft investigations “should include science packages to search for evidence of extinct or *extant* life” (ESA Cosmic Vision 2015–2025: http://www.esa.int/esapub/br/br247/br247.pdf; italics added); indeed the objectives of the agency's first Mars rover, ExoMars, are “to search for signs of past and *present* life on Mars” (http://exploration.esa.int/mars/45082-rover-scientific-objectives; italics added). And the NASA Astrobiology Institute includes, among its main objectives, the “search for evidence of ancient climates, extinct life and potential habitats for *extant* life on Mars” (https://nai.nasa.gov/media/roadmap/1998/objectives/o8_life_mars.html; italics added).

To check the consensus and priorities of the Mars community, a good start would be that a forthcoming MEPAG meeting put this question on the table. If we are erroneous, and the Mars community were to agree with Rummel and Conley (2017) that a mission to attempt detection of extant martian life is not a priority, then we, the Mars community, would have a clear mandate to straightforwardly convey that information to the global science community at large. We would need to send an unambiguous message to society that we are no longer interested in searching for currently active life on Mars and that the NASA Mars program would not pursue that objective in the foreseeable future. We are sure that will not be the outcome of this discussion. If we are correct, time is of the essence to start updating the policies related to the search for life on Mars: the Mars community needs to move forward toward a real Mars astrobiological exploration strategy within the next 2–3 decades. The benefits to science and to the global community at large, which would accompany such a pioneering search for life in the Solar System, are very much worth all our efforts today.

## References

[B1] Azua-Bustos A. and Vega-Martínez C. (2013) The potential for detecting ‘life as we don't know it’ by fractal complexity analysis. International Journal of Astrobiology 12:314–320

[B2] Benner S.A. (2017) Detecting Darwinism from molecules in the Enceladus plumes, Jupiter's moons, and other planetary water lagoons. Astrobiology 17:840–851 28665680 10.1089/ast.2016.1611 PMC5610385

[B3] Bhardwaj A., Sam L., Martín-Torres F.J., Zorzano M.P., and Fonseca R.M. (2017) Martian slope streaks as plausible indicators of transient water activity. Sci Rep 7, doi:10.1038/s41598-017-07453-9 PMC5539097 28765566

[B4] Carr C.E., Mojarro A., Hachey J., Saboda K., Tani J., Bhattaru S.A., Smith A., Pontefract A., Zuber M.T., Doebler R., and Brown M. (2017) Towards *in situ* sequencing for life detection. In 2017 IEEE Aerospace Conference, IEEE, Piscataway, NJ, doi:10.1109/AERO.2017.7943896

[B5] Cockell C., Catling D.C., Davis W.L., Snook K., Kepner R.L., Lee P., and McKay C.P. (2000) The ultraviolet environment of Mars: biological implications past, present, and future. Icarus 146:343–359 11543504 10.1006/icar.2000.6393

[B6] Conley C.A. and Rummel J.D. (2010) Planetary protection for human exploration of Mars. Acta Astronaut 66:792–797

[B7] Court R.W., Sims M.R., Cullen D.C., and Sephton M.A. (2014) Searching for life on Mars: degradation of surfactant solutions used in organic extraction experiments. Astrobiology 14:733–752 25192400 10.1089/ast.2013.1105

[B8] Dundas C.M., McEwen A.S., Chojnacki M., Milazzo M.P., Byrne S., McElwaine J.N., and Urso A. (2017) Granular flows at recurring slope lineae on Mars indicate a limited role for liquid water. Nat Geosci 10:903–907

[B9] Dybdahl M.F., Jenkins C.E., and Nuismer S.L. (2014) Identifying the molecular basis of host-parasite coevolution: merging models and mechanisms. Am Nat 184:1–13 24921596 10.1086/676591

[B10] Edwards C.S. and Piqueux S. (2016) The water content of recurring slope lineae on Mars. Geophys Res Lett 43, doi:10.1002/2016GL070179

[B11] Ehlmann B.L., Johnson S.S., Horgan B., Niles P.B., Amador E.S., Archer P.D. Jr, Byrne S., Edwards C.S., Fraeman A.A., Glavin D.P., Glotch T.D., Hardgrove C., Hayne P.O., Kite E.S., Lanza N.L., Lapotre M.G.A., Michalski J., Rice M., and Rogers A.D. (2017) Mars Exploration Science in 2050 [abstract 8236]. In Planetary Science Vision 2050 Workshop, Lunar and Planetary Institute, Houston, LPI Contribution 1989

[B12] ESA Cosmic Vision: Space Science for Europe 2015–2025, BR 247. (2005) ESA Publications Division, ESTEC, Noordwijk, The Netherlands

[B13] Fairén A.G. and Schulze-Makuch D. (2013) The overprotection of Mars. Nat Geosci 6:510–511

[B14] Fairén A.G., Parro V., Schulze-Makuch D., and Whyte L. (2017) Searching for life on Mars before it is too late. Astrobiology 17:962–970 28885042 10.1089/ast.2017.1703 PMC5655416

[B15] Fajardo-Cavazos P., Schuerger A.C., and Nicholson W.L. (2007) Testing interplanetary transfer of bacteria between Earth and Mars as a result of natural impact phenomena and human spaceflight activities. Acta Astronaut 60:534–540

[B16] Fox-Powell M.G., Hallsworth J.E., Cousins C.R., and Cockell C.S. (2016) Ionic strength is a barrier to the habitability of Mars. Astrobiology 16:427–442 27213516 10.1089/ast.2015.1432

[B17] Gordon P.R. and Sephton M.A. (2016) Organic matter detection on Mars by pyrolysis-FTIR: an analysis of sensitivity and mineral matrix effects. Astrobiology 16:831–845 27870586 10.1089/ast.2016.1485 PMC5124741

[B18] Grossman L. (2013, 7 20) NASA urged to seek live martians with 2020 rover. New Sci 219:9

[B19] Hand E. (2018) Mars methane rises and falls with the seasons. Science 359:16–17 29301992 10.1126/science.359.6371.16

[B20] Heldmann J.L., Schurmeier L., McKay C., Davila A., Stoker C., Marinova M., and Wilhelm M.B. (2014) Midlatitude ice-rich ground on Mars as a target in the search for evidence of life and for *in situ* resource utilization on human missions. Astrobiology 14:102–118 24506507 10.1089/ast.2013.1103

[B21] Hubbard S. (2017) Keeping the focus on Mars. New Space 5:201–202

[B22] Jonkers H.M., Thijssen A., Muyzer G., Copuroglu O., and Schlangen E. (2010) Application of bacteria as self-healing agent for the development of sustainable concrete. Ecol Eng 36:230–235

[B23] Khodadad C.L., Wong G.M., James L.M., Thakrar P.J., Lane M.A., Catechis J.A., and Smith D.J. (2017) Stratosphere conditions inactivate bacterial endospores from a Mars spacecraft assembly facility. Astrobiology 17:337–350 28323456 10.1089/ast.2016.1549 PMC5399745

[B24] King G.M. (2015) Carbon monoxide as a metabolic energy source for extremely halophilic microbes: implications for microbial activity in Mars regolith. Proc Natl Acad Sci USA 112:4465–4470 25831529 10.1073/pnas.1424989112 PMC4394273

[B25] La Duc M.T., Dekas A., Osman S., Moissl C., Newcombe D., and Venkateswaran K. (2007) Isolation and characterization of bacteria capable of tolerating the extreme conditions of clean room environments. Appl Environ Microbiol 73:2600–2611 17308177 10.1128/AEM.03007-06 PMC1855582

[B26] Levin G.V. and Straat P.A. (2016) The case for extant life on Mars and its possible detection by the Viking Labeled Release Experiment. Astrobiology 16:798–810 27626510 10.1089/ast.2015.1464 PMC6445182

[B27] Martín-Torres J. and Zorzano M.P. (2017) Should we invest in martian brine research to reduce Mars exploration costs? Astrobiology 17:3–7 28026989 10.1089/ast.2016.1602 PMC5278815

[B28] Mattingly R., Matousek S., and Gershman R. (2002) Mars sample return—studies for a fresh look. In Aerospace Conference Proceedings, 2002, Vol. 2, IEEE, Piscataway, NJ, doi:10.1109/AERO.2002.1035551

[B29] May R.M. and Anderson R.M. (1990) Parasite–host coevolution. Parasitology 100:S89–S101 2122393 10.1017/s0031182000073042

[B30] McKay D.S., Gibson E.K. Jr, Thomas-Keprta K.L., and Vali H. (1996) Search for past life on Mars: possible relic biogenic activity in martian meteorite ALH84001. Science 273:924–930 8688069 10.1126/science.273.5277.924

[B31] McKay C.P., Stoker C.R., Glass B.J., Davé A.I., Davila A.F., Heldmann J.L., Marinova M.M., Fairen A.G., Quinn R.C., Zacny K.A., and Paulsen G. (2013) The Icebreaker Life Mission to Mars: a search for biomolecular evidence for life. Astrobiology 13:334–353 23560417 10.1089/ast.2012.0878

[B32] MEPAG. (2015) Mars Science Goals, Objectives, Investigations, and Priorities, posted 6 2015 by the Mars Exploration Program Analysis Group (MEPAG). Available online at https://mepag.jpl.nasa.gov/reports/CombinedGoals_June18_FINAL.pdf

[B33] Michalski J.R., Onstott T.C., Mojzsis S.J., Mustard J., Chan Q.H., Niles P.B., and Johnson S.S. (2018) The Martian subsurface as a potential window into the origin of life. Nat Geosci 11:21–26

[B34] Mileikowsky C., Cucinotta F.A., Wilson J.W., Gladman B., Horneck G., Lindegren L., Melosh J., Rickman H., Valtonen M., and Zheng J.Q. (2000) Natural transfer of viable microbes in space: 1. From Mars to Earth and Earth to Mars. Icarus 145:391–427 11543506 10.1006/icar.1999.6317

[B35] Mojarro A., Ruvkun G., Zuber M.T., and Carr C.E. (2017) Nucleic acid extraction from synthetic Mars analog soils for *in situ* life detection. Astrobiology 17:747–760 28704064 10.1089/ast.2016.1535 PMC5567878

[B36] Niles P.B., Beaty D., Hays L., Bass D., Bell M.S., Bleacher J., Cabrol N.A., Conrad P., Eppler D., Hamilton V., Head J., Kahre M., Levy J., Lyons T., Rafkin S., Rice J., and Rice M. (2017) Scientific investigations associated with the human exploration of Mars in the next 35 years [abstract 8167]. In Planetary Science Vision 2050 Workshop, Lunar and Planetary Institute, Houston

[B37] Ojha L., Wilhelm M.B., Murchie S.L., McEwen A.S., Wray J.J., Hanley J., Massé M., and Chojnacki M. (2015) Spectral evidence for hydrated salts in recurring slope lineae on Mars. Nat Geosci 8:829–832

[B38] Parro V., de Diego-Castilla G., Rodriguez-Manfredi J.A., Rivas L.A., Blanco-Lopez Y., Sebastian E., Romeral J., Compostizo C., Herrero P.L., Garcia-Marin A., Moreno-Paz M., Garcia-Villadangos M., Cruz-Gil P., Peinado V., Martin-Soler J., Perez-Mercader J., and Gomez-Elvira J. (2011) SOLID3: a multiplex antibody microarray-based optical sensor instrument for *in situ* life detection in planetary exploration. Astrobiology 11:15–28 21294639 10.1089/ast.2010.0501

[B39] Rummel J.D. (2009) Special regions in Mars exploration: problems and potential. Acta Astronaut 64:1293–1297

[B40] Rummel J.D. and Conley C.A. (2017) Four fallacies and an oversight: searching for martian life. Astrobiology 17:971–974 28920443 10.1089/ast.2017.1749 PMC5655418

[B41] Rummel J.D., Beaty D.W., Jones M.A., Bakermans C., Barlow N.G., Boston P.J., Chevrier V.F., Clark B.C., de Vera J.P.P., Gough R.V., Hallsworth J.E., Head J.W., Hipkin V.J., Kieft T.L., McEwen A.S., Mellon M.T., Mikucki J.A., Nicholson W.L., Omelon C.R., Peterson R., Roden E.E., Sherwood Lollar B., Tanaka K.L., Viola D., and Wray J.J. (2014) A new analysis of Mars “Special Regions”: findings of the second MEPAG Special Regions Science Analysis Group (SR-SAG2). Astrobiology 14:887–968 25401393 10.1089/ast.2014.1227

[B42] Schuerger A.C. and Nicholson W.L. (2006) Interactive effects of hypobaria, low temperature, and CO_2_ atmospheres inhibit the growth of mesophilic *Bacillus* spp. under simulated martian conditions. Icarus 185:143–152

[B43] Schulze-Makuch D. and Davila A.F. (2010) Searching for life beyond our planet: are we there yet? Eos 91:280

[B44] Schulze-Makuch D., Fairén A.G., and Davila A. (2013) Locally targeted ecosynthesis: a proactive *in situ* search for extant life on other worlds. Astrobiology 13:674–678 23848472 10.1089/ast.2013.0995

[B45] Schulze-Makuch D., Rummel J.D., Benner S.A., Levin G., Parro V., and Kounaves S. (2015) Nearly forty years after Viking: are we ready for a new life-detection mission? Astrobiology 15:413–419 26053735 10.1089/ast.2015.1336

[B46] Smith J.P., Smith F.C., and Booksh K.S. (2017) Spatial and spectral resolution of carbonaceous material from hematite (α-Fe_2_O_3_) using multivariate curve resolution-alternating least squares (MCR-ALS) with Raman microspectroscopic mapping: implications for the search for life on Mars. Analyst 142:3140–3156 28678223 10.1039/c7an00481h

[B47] Vago J., Witasse O., Svedhem H., Baglioni P., Haldemann A., Gianfiglio G., Blancquaert T., McCoy D., and de Groot R. (2015) ESA ExoMars program: the next step in exploring Mars. Solar System Research 49:518–528

[B48] Wadsworth J. and Cockell C.S. (2017) Perchlorates on Mars enhance the bacteriocidal effects of UV light. Sci Rep 7, doi:10.1038/s41598-017-04910-3 PMC5500590 28684729

[B49] Woolhouse M.E., Webster J.P., Domingo E., Charlesworth B., and Levin B.R. (2002) Biological and biomedical implications of the co-evolution of pathogens and their hosts. Nat Genet 32:569–577 12457190 10.1038/ng1202-569

[B50] Woolman P.F., Pearson V.K., Cockell C.S., and Olsson-Francis K. (2015) Subsurface halophiles: an analogue for potential life on Mars. In Life on Earth and Beyond: The History and Philosophy of the Origin of Life, 4–6 5 2015, Ven Island, Sweden

[B51] Xie T., Mittelholz A., and Osinski G.R. (2017) The use of Raman spectroscopy for the 2016 CanMars MSR analogue mission [abstract 1581]. In 48^th^ Lunar and Planetary Science Conference, Lunar and Planetary Institute, Houston

